# Endometriotic Mesenchymal Stem Cells Epigenetic Pathogenesis: Deregulation of *miR-200b, miR-145,* and *let7b* in A Functional Imbalanced Epigenetic Disease

**DOI:** 10.22074/cellj.2019.5903

**Published:** 2019-02-25

**Authors:** Parisa Mashayekhi, Mehrdad Noruzinia, Sirous Zeinali, Sepideh Khodaverdi

**Affiliations:** 1 Department of Medical Genetics, Faculty of Medical Sciences, Tarbiat Modares University, Tehran, Iran; 2 Biotechnology Research Center, Pasteur Institute of Iran, Tehran, Iran; 3 Endometriosis Research Center, Iran University of Medical Science, Tehran, Iran

**Keywords:** Cell Differentiation, Cell Self-Renewal, Mesenchymal Stromal Cells, microRNAs

## Abstract

**Objective:**

Stem cell issue is a strong theory in endometriosis pathogenesis. It seems that endometriotic mesenchymal stem cells (MSCs) show different characteristics compared to the normal MSCs. Determined high proliferation and low differentiation/ decidualization potential of endometriotic MSCs could be accompanied by their microRNAs deregulation influencing their fate and function. In this study for the first time, we evaluated the expression of *miR-200b, miR-145,* and *let7b* in endometriotic compared to non-endometriotic MSCs. These microRNAs are involved in biological pathways related to proliferation and differentiation of stem cells. Their aberrant expressions can disturb the proliferation/ differentiation balance in stem cells, altering their function and causing various diseases, like endometriosis.

**Materials and Methods:**

In this experimental study, MSCs were isolated from three endometriotic and three non- endometriotic eutopic endometrium, followed by their characterization and culture. Expression of *miR-200b, miR-145,* and *let-7b* was ultimately analyzed by quantitative reverse transcription polymerase chain reaction (qRT-PCR).

**Results:**

We found that the expression of *miR-200b* was up-regulated (P<0/0001) whereas the expression of miR- 145 and let-7b was down-regulated (P<0.0001) in endometriotic MSCs in comparison with non-endometriotic normal controls.

**Conclusion:**

Proliferation and differentiation are important dynamic balanced biological processes, while in equillibrium, they determine a healthy stem cell fate. It seems that they are deregulated in endometriotic MSCs and change their function. *miR-200b, miR-145,* and *let7b* are deregulated during endometriosis and they have pivotal roles in the modulating proliferation and differentiation of stem cells. We found up-regulation of miR-200b and down-regulation of *miR-145* and *let-7b* in endometriotic MSCs. These changes can increase self-renewal and migration, while decreasing differentiation of endometriotic MSCs. Our achievements emphasize previous findings on the importance of proliferation/ differentiation balance in MSCs and clarify the role of microRNAs as main players in faulty endometriotic stem cells development.

## Introduction

Endometriosis is a common, benign gynecologic disorder recognized by the presence of the endometrial tissue out of the uterus, especially on pelvic organs and peritoneum. The most clinical presentation is pelvic pain worsen during menstruation, painful intercourse and infertility. Endometriosis affects approximately 10% of women in reproductive age and it may occur in about 50% of those with pelvic pain, infertility or both (1).

Several theories have thus far been proposed including retrograde menstruation, coelomic metaplasia, steroid hormones, oxidative stress, impaired immune function, decreased apoptosis, genetics, epigenetics, and stem cells, while evidences show each of these factors has partially been involved in endometriosis pathogenesis (2).

During each menses, almost all of the functional layer and small amount of the basalis layer containing a lot of stem cells shed in the uterus (3). They can migrate out of the uterus through retrograde menstruation, seed there and establish endometriotic lesions. However, the presence of endometriosis in 10% of women despite the presence of retrograde menstruation in over 90% of them seems intriguing. Several evidences show that the stem cells generating endometriotic lesions are characteristically different from the normal stem cells. They have a higher ability to proliferate and a lower capacity for differentiation and decidualization (4). It appears to us that impaired proliferation/differentiation and decidualization balance can changes stem cell character and function, while this makes them susceptible to develop endometriosis.

Several studies investigated genetic contribution in endometriosis, most of which failed to determine any significant correlation. Some studies demonstrated that epigenetic deregulation is, in fact, the underlying pathogenic mechanism of endometriosis (5) and it alters gene expression in response to hormonal and environmental factors (i.e., through dynamic changes of the environment). 

Epigenetic changes play an important role in the pathogenesis of various diseases, including cancers, and they are used as biomarkers for early diagnosis (6). Epigenetics is longtime proved concept, involved in stem cell regulation (7). microRNAs (miRs) are short non-coding RNA molecules with critical roles in posttranscriptional regulation of different genes (8) and, as epigenetic regulators, they are key molecules involved in the determination of stem cell fate by regulation of the self- renewal and differentiation-related pathways (9). Their aberrant expression can change stem cell functions and cause the differences between endometriotic and normal stem cells (10). Thus far, deregulation of microRNAs has been confirmed to contribute to endometriosis and infertility (11).

In this study, we chose three microRNAs (*miR-200b, miR-145* and *let-7b*) dysregulated during endometriosis (12) and their expressions were evaluated in endometriotic mesenchymal stem cells (MSCs).

Aberrant expression of *miR-200b* has been reported in many cancers (13). Up-regulation of this microRNA promotes cell proliferation in cervical cancer (14). Transfection of endometriotic stem cells with *miR-200b* increases cell proliferation and side population phenotype through enhancing expression of *KLF4, SOX2, OCT4* and *c-MYC*, in addition to transforming mature cells into pluripotent cells (15). *miR-200c* overexpression in human embryonic stem cells (hESCs) up-regulates *NANOG* expression and decreases apoptosis, resulting in maintenance of their self-renewal ability and proliferation (16). *miR-200* family helps transition of human fibroblasts to pluripotent stem cells by *ZEB2* suppression and mesenchymal-epithelial transition (MET) induction in cooperation with *OCT4* and *SOX2* (17). 

Overexpression of *miR-145* inhibits cell proliferation and migration by suppressing the *TGF-ß1* expression in breast cancer cells (18). This microRNA induces differentiation of cervical cancer stem cells (CSCs) by suppressing the stem cell transcription factors involved in maintaining CSCs self-renewal (19). miR-145 acts as a tumor suppressor molecule in a lot of cancers (20). miR-145 inhibits endometriotic cell proliferation, and self-renewal via targeting *OCT4, KLF4,* and *SOX2* and induces hESC and CSCs differentiation (21-23). Its expression is down-regulated in hESCs and increased within differentiation. 

*let-7* is strongly accepted as a tumor suppressor microRNA and expression of its family members are down-regulated in several types of cancer (24). *let-7b* suppresses the expression of *OCT4* as well as SOX2 and it reprogrammes CSCs into the differentiated cells via a *let-7/LIN28* feedback loop (25). *let-7b* overexpression inhibits proliferation and induces differentiation in adult and CSCs (26). 

It seems that *miR-200b, miR-145,* and *let7b* could be involved in the modulation of self-renewal and differentiation of stem cells, so their role in stem cell dysfunction could be postulated as a plausible theory.

Considering this hypothesis, we compared the expression of these microRNAs (*miR-200b, miR-145,* and *let-7b*) in MSCs isolated from three women who had pelvic endometriosis and three women without endometriosis. This comparison shows the aberrant expression of these microRNAs in endometriotic MSCs and supports the presence of proliferation/differentiation imbalance in endometriosis initiating MSCs.

## Materials and Methods

### Ethics statement

This study was approved by the Ethics Committee of Medical Faculty of Tarbiat Modares University (no. 1395.409), Tehran, Iran. Written informed consent was taken from each patient after a standard genetic counselling. 

### Specimen sources

Human endometrial tissue samples were obtained from three premenopausal women (30-45 years old) undergoing hysterectomy for non-endometrial benign pathological condition and another three patients with endometriosis undergoing laparoscopy for endometriosis in the Rasoul Akram Hospital of Iran Medical University (Tehran, Iran). Eutopic endometrial tissues were obtained from the patients. The patients had not received hormone treatments for at least three months before sample collection. Diagnosis of endometriotic and non-endometriotic collected tissues was validated by histopathological test by two experienced histopathologists. 

### Isolation and culture of human endometrial mesenchymal stem cells

Tissues were separated and washed in phosphate buffered saline (PBS) then minced into 1-2 mm3 pieces in a medium containing Dulbecco modified Eagle medium/ Ham’s F-12 (DMEM/F-12, Invitrogen, UK) and 1% penicillin-streptomycin antibiotics solution (Invitrogen, USA). Briefly, cell suspension of endometrial cells was obtained using enzymatic digestion using collagenase type 3 (300 µg/ml, Sigma, Germany) and mechanical procedure at 37°C for 90 minutes, then centrifuged for 5 minutes at 3000 rpm. Cell suspensions were filtered through 150, 100, 40 mm mesh to remove undigested tissues and epithelial components. Endometrial stromal cells were next cultured in DMEM/F-12 containing 1% penicillin-streptomycin solution and 10% fetal bovine serum (FBS, Gibco, USA) at 37°C in 95% air and 5% CO_2_ conditions. Endometrial stromal cells in passages 3-4 were used for characterization by flow cytometry analyses. 

### Endometrial stromal cells flow cytometry analysis 

Isolated stromal cells were trypsinized and centrifuged. The cell pellet was resuspended in PBS supplemented with 5% FBS and incubated with monoclonal antibodies for 30 minutes at 4°C in the dark. Human CD45 (BD Bioscience, USA) and CD34 (IMMUNOSTEP, Spain) antibodies were served as negative controls, while anti-human CD90 (BD Bioscience, USA), CD105 (IMMUNOSTEP, Spain), CD73 (BD Bioscience, USA) and CD146 (BD Bioscience, USA) were used as specific antibodies. Cells were evaluated with a FACS Calibur apparatus (Becton Dickinson, USA). Finally, the analysis was done using FlowJo 7.6 software. 

### Differentiation of endometrial mesenchymal stem cells 

For evaluating the endometrial MSCs differentiation potential, endometrial stromal cells (CD146+, CD90+, CD105+, CD73+ and CD34-, CD45-) were seeded in 24-well plates and cultured in osteogenic and adipogenic differentiation media for 4 weeks, separately. Control cells were also cultured in low serum medium (DMEM/F12 with 1% FBS and 1% penicillin-streptomycin antibiotic solution) for the same incubation time. Control and differentiation media were changed every 2-3 days. Three weeks later, osteogenic and adipogenic differentiations were respectively checked by staining with 4% Alizarin Red (pH=4.1) and 1% Oil Red O (both from Sigma, Germany) (27). 

### RNA extraction and cDNA synthesis 

Total RNA was extracted from the cells using TRIzol reagent (Sigma, Germany). RNA concentration and purity were assessed by Nanodrop (the ratio of absorbance at 260 and 280 nm =1.8), then we ran the extracted RNA on denaturing agarose gel electrophoresis and the gel was stained with ethidium bromide for evaluating the quality of extracted RNA. cDNA was synthesized using specific stem-loop primers for microRNAs (*miR-200b, let-7b, miR-145, * and *RNU44*) in a total volume of 20 µl using the cDNA synthesis kit (Takara Bio, Japan). 

Stem-loop RT primers were designed in accordance with the protocol described by Chen et al. (28). Primer sequences are presented in Table 1.

### Quantitative reverse transcription polymerase chain reaction for evaluation of the microRNA expression levels 

To determine expression of the microRNAs (*miR-200b, miR-145* and *let-7b*) in the cells, we used the Allele ID6 and Oligo7 software for designing the specific forward primers and universal reverse primer. RNU44 was used as an internal control. Primers were synthesized at Pishgam Co. (Tehran, Iran). We used Syber Green Assay kit (Applied Biosystems, UK) according to the manufacturer’s protocol. qRT-PCR reactions were done in 10 µl of the reaction mixture using AB StepOne Real- Time PCR System (Applied Biosystems, UK). All qRT- PCR experiments were repeated three times. Data were analyzed using Pfaffl method and normalized by *RNU44* expression in each sample. 

### Statistical analysis 

We used student’s t test by GraphPad Prism 6 software for statistical analysis and comparison of microRNA expressions between samples. Results were considered significant at P<0.05. 

## Results

### Isolation and characterization of endometrial mesenchymal stem cells 

**Fig.1:**
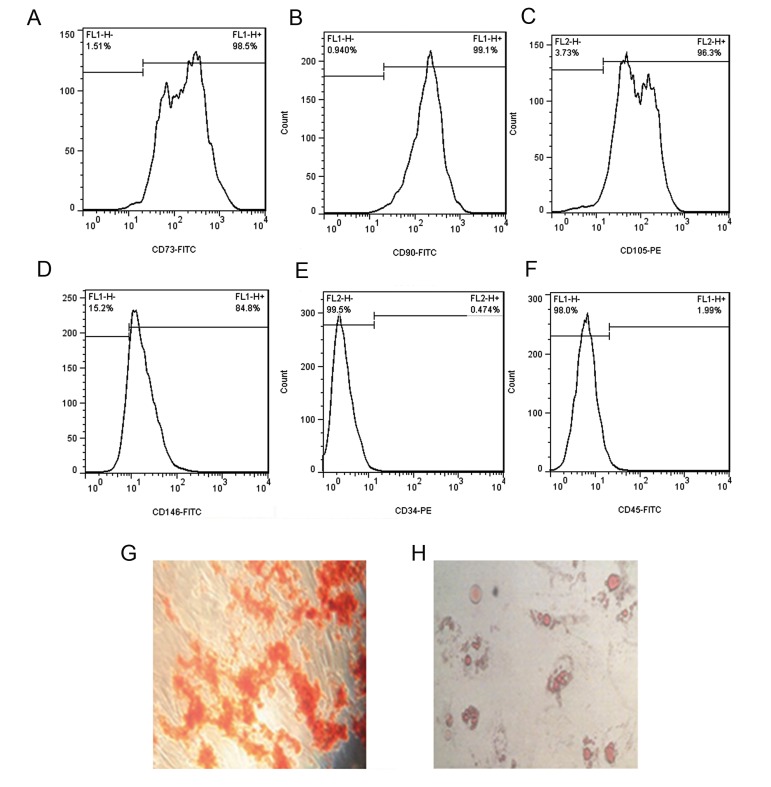
Isolation and characterization of endometrial mesenchymal stem cells (MSCs). Flow cytometry analyses showed that endometrial MSCs positively expressed **A.** CD73 (98.5%), **B.** CD90 (99.1%), **C.** CD105 (96.3%), **D.** CD146 (84.9%) but negatively expressed, **E.** CD34 (0.474%), **F.** CD45 (1.99%), G. Osteogenic, and **H.** adipogenic differentiation of the isolated endometrial MSCs.

Human MSCs were isolated from the endometrium and they were cultured. Flow cytometry analysis confirmed the expression of MSC markers CD73 (98.5%), CD90 (99.1%), CD105 (96.3%) and CD146 (84.8%). Expression of hematopoietic markers, including CD34 (0.474%) and CD45 (1.99%), were negative (Fig .1A-F). To evaluate differentiation potential of the isolated endometrial MSCs, we induced adipogenic and osteogenic differentiation with specific differentiation media, as specified. Confirmation of differentiation was done through staining of calcium deposits by alizarin red and lipid vacuoles through oil red staining (Fig .1G, H). 

**Table 1:** Sequence of oligonucleotide primers used for quantitative reverse transcription polymerase chain reaction (qRT-PCR) measurements


Primer name	Sequence (5´-3´)

*let-7b stem-loop primer*	GTCGTATCCAGTGCAGGGTCCGAGGTATTCGCACTGGATACGACAACCAC
*let-7b forward primer*	GCTCTTGAGGTAGTAGGTTGTGTG
*miR-200b stem-loop primer*	GTCGTATCCAGTGCAGGGTCCGAGGTATTCGCACTGGATACGACTCATCA
*miR-200b forward primer*	CGCTAATACTGCCTGGTAATGATGA
*miR-145 stem-loop primer*	GTCGTATCCAGTGCAGGGTCCGAGGTATTCGCACTGGATACGACAGGGAT
*miR-145 forward primer*	CATCCGTCCAGTTTTCCCAGG
*RNU44 stem-loop primer*	GTCGTATCCAGTGCAGGGTCCGAGGTATTCGCACTGGATACGACAGTCAG
*RNU44 forward primer*	TCACGCCTGGATGATGATAAGC
*Universe reverse primer*	CAGTGCAGGGTCCGAGGTA


### Quantitative reverse transcription polymerase chain reaction 

To explore microRNAs profiling in endometrial MSCs of the endometriotic and non-endometriotic control groups the expression levels of *miR-200b, miR-145* and *let-7b* were evaluated by qRT-PCR. The efficiency of qRT-PCR reactions for *miR-200b, miR-145* and *let-7b* were measured using LinReg software algorithm (29). Each experiment was repeated three times to eliminate any subjective variation. All reactions were assessed for distinct melting curves, while they showed no nonspecific or primer-dimer peaks. 

### miR-200b was up-regulated in endometriotic mesenchymal stem cells

**Fig.2:**
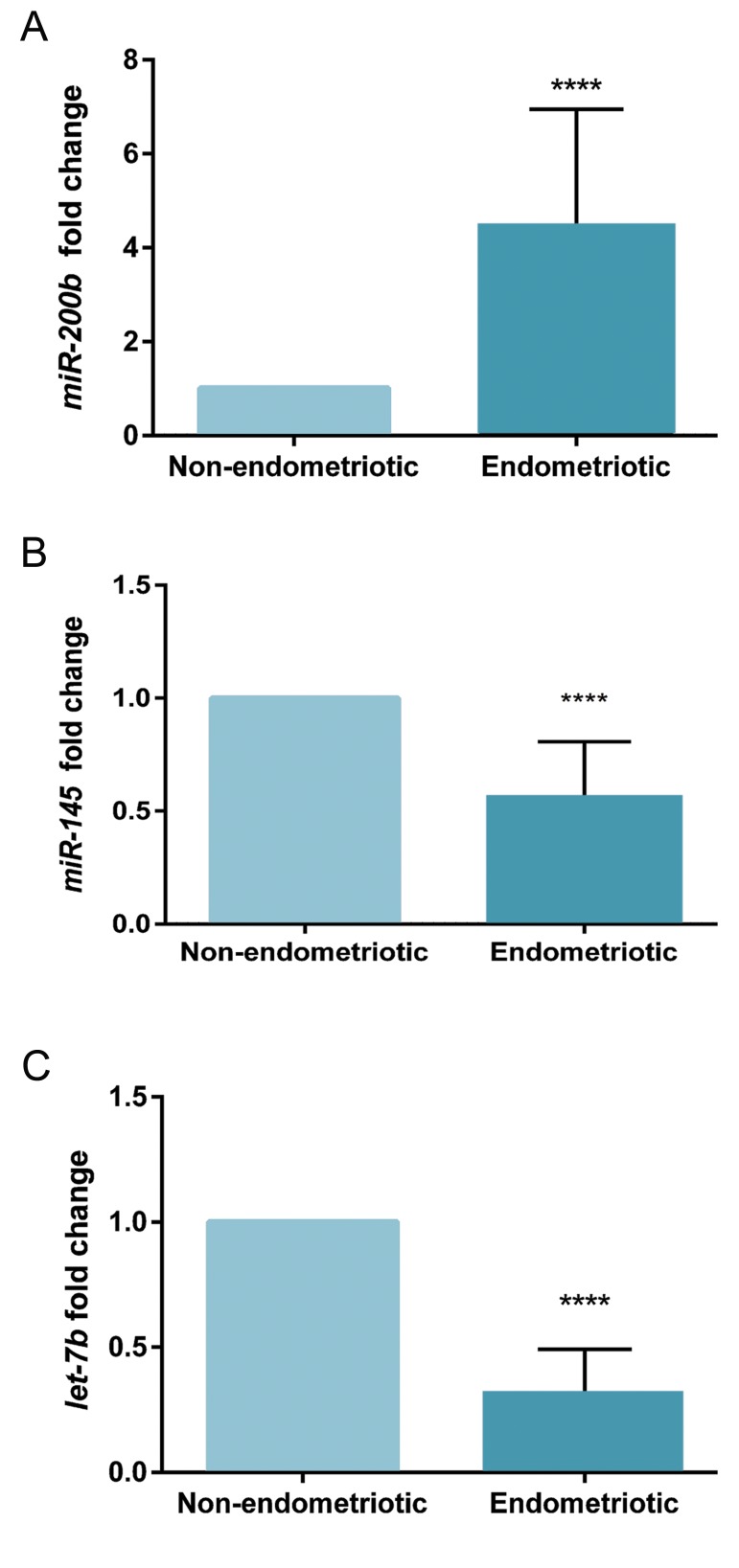
microRNA expression analyses. Relative expressions of miR200b, miR-145 and let-7b in endometrial mesenchymal stem cells (MSCs) of endometriotic patients and non-endometriotic control group, evaluated by quantitative reverse transcription polymerase chain reaction (qRT-PCR). A. miR-200b expression in endometritic MSCs was 4.199 ± 0.6617 fold (P<0.0001) higher than nonendometriotic MSCs, B. miR-145 expression in endometritic MSCs was 0.5467 ± 0.06137 fold (P<0.0001) less than non-endometriotic MSCs, and C. Expression of let-7b in endometriotic MSCs was 0.3024 ± 0.04454 fold (P<0.0001) less than non-endometriotic MSCs control group. ****; P<0.0001 in comparison to non-endometriotic MSCs.

Relative expressions of miR-200b in the endometriotic MSCs showed up-regulation of this microRNA (4.199 ± 0.6617, P<0.0001) in comparison with the nonendometriotic control group (Fig .2A).

### *miR-145* was down-regulated in endometriotic mesenchymal stem cells 

Expression of miR-145 in the endometriotic MSCs was decreased to 0.5467 ± 0.06137 fold (P<0.0001) in comparison with the non-endometriotic control group (Fig .2B). 

### let-7b was down-regulated in endometriotic mesenchymal stem cells

Expression of *let-7b* in the endometriotic MSCs was 0.3024 ± 0.04454 fold (P<0.0001) less than the nonendometriotic control group (Fig .2C).

## Discussion

We believe that proliferation/differentiation imbalance plays a pivotal role in the pathogenesis of endometriosis. We evaluated *miR-200b, miR-145* and *let-7b* expression as modulators of stem cell proliferation and differentiation (30), in endometriotic and non-endometriotic MSCs. Previous studies have shown that these microRNAs were deregulated in endometriosis while no study evaluated their expression in endometriotic MSCs (31, 32). 

Several theories are proposed as the pathogenesis basis of endometriosis. stem cell theory is one main research field in endometriosis. A lot of studies described the role of stem cells in endometriosis development (3). A balance in proliferation/differentiation equilibrium is required for the correct function of stem cells and it seems to us that in several diseases including endometriosis, this balance fails, resulting in altered function of stem cells, and changing their fate. Many studies confirm that endometriotic MSCs are different from non-endometriotic types. They have a higher ability to migrate, attach and proliferate (33, 34), while a lower capacity for differentiation and decidualization is proposed for them, due to the impaired decidualization related pathways (4). We believe that proliferation/differentiation imbalance in endometriotic MSCs is the main underlying cause for endometriosis development and its correlated infertility. 

Previous studies have shown that microRNAs are involved in regulation of signaling pathways that control differentiation and proliferation of stem cells during normal development and disease pathogenesis (30). 

*miR-200b, miR-145* and *let-7b* are deregulated in several diseases like cancers confirming the aforementioned imbalance. These microRNAs have specific expression profile in endometrial stromal cells during decidualization (35). Deregulation of these microRNAs has been shown in the ectopic and eutopic endometrium of women with endometriosis, but our study is the first to confirm their expressions and roles in endometriotic MSCs. We find that in endometriotic MSCs *miR-200b* is up-regulated significantly as compared to normal control group. Previous studies have shown that *miR-200b* is up-regulated in eutopic endometrium of endometriotic women and involved in endometriosis-associated infertility (31). Overexpression of *miR-200b* increases cell proliferation and MET. It induces generation of pluripotent stem cells in cooperation with transcription factor *SOX2* and *OCT4* (17).

Transfection of endometriotic stem cells with *miR-200b* results in increase side population phenotype through activating *KLF4* and *NANOG* expressions as well as MET, while reducing decidualization. It also enhances metastatic colonization of successfully migrated cells by inhibiting secretion of metastasis inhibitors (15). *miR200* family members are down-regulated during *in vitro* decidualization (35). 

Increased expression of *miR-200b* in endometriotic MSCs, in our study, is in accordance with the findings of previous studies. It might increase colonization chance of the migrated stem cells, enhance their proliferation and promote their stemness properties by positive regulation of stemness-related genes while decreasing the differentiation potential and decidualization. These changes promote development of endometriosis, disrupt embryo implantation and cause infertility. 

Our findings show that *miR-145* is down-regulated in endometriotic MSCs. Previous studies have demonstrated that *miR-145* is down-regulated in the serum of endometriotic patients in comparison with normal control and potentially served as noninvasive biomarkers for endometriosis. Transfection of endometrial stromal cells with *miR-145* inhibits cell proliferation and invasiveness. It also suppresses the stemness by down-regulation of stemness-related genes (36). This microRNA induces differentiation of stem cells through SOX2-LIN28/let7 signaling pathway by decreasing SOX2 and LINE-28 protein levels (37). Overexpression of this microRNA in CSCs reduces the expression of stemness-related markers, while it increases cancer cells differentiation (38). In the present study, decreased level of *miR-145* in endometriotic MSCs confirms findings obtained from previous studies. This is consistent with the underlying proposed pathogenesis mechanism to increase stem cell proliferation, decrease their differentiation and facilitate endometriosis risk. 

Our results show a down-regulation of *let-7b* in the endometrial MSCs of women with endometriosis. Previous studies have also shown that expression of *let-7* was decreased in the serum of endometriotic patients in comparison with normal control (32).

*let-7* is involved in a regulatory feedback loop with LIN28, which has a critical role in pluripotency maintenance in collaboration with *NANOG, SOX2* and *OCT4* genes. Overexpression of this microRNA in stem cells promotes differentiation, while inhibition of *let-7* results in the proliferation of stem cells and decreases differentiation. Briefly, *let-7b* family members fine- tune the pathways related to self-renewal/differentiation balances (39). 

*let-7* suppresses the expression of *OCT4* and *SOX2*. It reprogrammes CSCs to differentiate via *let-7/LIN28* feedback loop and its overexpression regulates the stemness by increasing differentiation and decreasing self-renewal in both of the normal and cancer stem cells (26) Reduced level of *let-7* is required for self- renewal and maintenance of the undifferentiated state of embryonic and adult stem cells and its overexpression has opposing effects, reducing their proliferation and leading to their differentiation (39). Overexpression of *let-7b* in neural stem cells inhibits proliferation and promotes differentiation (40). 

In this study, *let-7b* down-regulation in endometriotic MSCs consolidates the results of previous studies. *let- 7b* is proposed as one of the main players of proliferation/ differentiation imbalance in endometriotic MSCs. In other words, any deregulation of *let-7b* expression alters proliferation/differentiation balance in endometriotic MSCs. *let-7b* deregulation increases the probability of endometriotic lesion formations via enhancing the stem cell proliferation, migration, self-renewal and maintenance of their undifferentiated state. These changes reduce decidualization and increase infertility in patients with endometriosis. 

Although the exact underlying pathologic mechanism of endometriosis is yet unclear, current findings discover the strong role of stem cells in endometriosis and confirm their different characteristics and function. Our results consolidate the theory of imbalance between differentiation and proliferation capacity, especially in stem cells of endometriotic patients. This study for the first time evaluates the expression of *miR-200b, miR-145* and *let-7b* in endometrial MSCs of women with endometriosis in comparison with normal control, representing that aberrant expression of these microRNAs is present in this pathological condition. These microRNAs contribute to modulating proliferation and/or differentiation of stem cells. This is the first study to evaluate the expression of these microRNAs in endometriotic stem cells. Our findings are in support of a unified differentiation/proliferation imbalance theory. 

## Conclusion

Endometriosis is a complex and yet unknown gynecological disease in women. Deregulation of microRNAs related to differentiation and proliferation in endometriotic MSCs compared to the normal types confirms the implication of epigenetics and this is in line with many other authors, while supporting the underlying mechanism of endometriosis, as emphasized in this study. We think that impaired balance between differentiation and proliferation in MSCs, which is supported by our study, is essential for endometriosis development.
